# Hospitality education evolution observed in online learning dataset

**DOI:** 10.1016/j.dib.2022.108525

**Published:** 2022-08-08

**Authors:** Kang-Lin Peng, Jusi Xu, Xin Wang, Huawen Shen

**Affiliations:** a Faculty of International Tourism and Management, City University of Macau, Macau SAR, China; b School of Hospitality and Tourism Management, Shunde Polytechnic, Shunde District, Foshan City, Guangdong Province, China

**Keywords:** COVID-19 pandemic, education mutation, hybrid-learning, information entropy, real-time online courses data

## Abstract

The research article "Realtime Online Courses Mutated amid the COVID-19 Pandemic: Empirical Study in Hospitality Program" aims to explore the education evolution amid the pandemic [1]. Data were collected by recruiting 956 respondents; 926 responses were adopted after the valid screening through a cooperative survey company. A random sampling of targeted groups was required when outsourcing the data collection to the survey company Wenjuanxing, a platform with a majority population database providing functions equivalent to Amazon Mechanical Turk [2]. We asked the company to deliver the designed questionnaire to teachers and students in hospitality programs. The reliability and validity of all constructs showed that the questionnaire is proper for measurement [3]. Data analysis applied the structural equation model with Mplus to examine the CFA model and research hypothesis. Structural equation modeling was applied to conduct the hypotheses test and model fitness through the statistical tool Mplus. Results imply that the data is suitable for conducting replication studies.

## Specifications Table

**Table:** 

Subject	Tourism, Leisure and Hospitality Management
Specific subject area	Hospitality education is primarily about knowledge of hotels, bars, and restaurants operations. The area often overlaps with Tourism which focuses on psychological satisfaction.
Type of data	Table
How the data were acquired	Survey (the supplementary material is attached) The questionnaire is designed through literature review and adjusted by reliability and validity tests.
Data format	Raw
Description of data collection	Data were collected by recruiting 956 respondents; 926 responses were adopted after the valid screening through a cooperative survey company. A random sampling of targeted groups was required when outsourcing the data collection to the survey company Wenjuanxing, a platform with a majority population database providing functions equivalent to Amazon Mechanical Turk [2]. We asked the company to deliver the designed questionnaire to teachers and students in hospitality programs through a random sampling approach in their population database. The reliability and validity of all constructs showed that the questionnaire is proper for measurement [4].
Data source location	Region: Chinese Mainland, Hong Kong SAR, Macau SAR, Taiwan
Data accessibility	Repository name: Mendeley Data Direct URL to data: https://doi.org/10.17632/6gjyr6vm9g.1
Related research article	K.-L. Peng, P. M. C. Lin, J. Xu, and X. Wang, Realtime online courses mutated amid the COVID-19 pandemic: Empirical study in the hospitality program, *Journal of Hospitality, Leisure, Sport & Tourism Education,* 30 (2022) 100379. https://doi.org/10.1016/j.jhlste.2022.100379[1].

## Value of the Data

• The data is useful because it is applied to the research model we have done and could also extend the model with more constructs to conduct the competitive statistical models. The measurement scales of learning outcomes could apply different analysis methods because the scales were developed into two various forms. One form is Bloom's Taxonomy of the Cognitive Domain, with six levels of learning outcome in a question; the other question item measures six levels individually. We conducted SEM to test the model using the later form. One might use regression analysis to test the cause-effect between two constructs. For example, a regression analysis from the cause construct “social media functionality” to the effect construct “learning outcome” applied the former form.
• Professors can also use the data to illustrate a course in statistical exploitation of survey data that focuses on structural equation modeling. The scientific and pedagogical orientations can be highlighted in teaching and learning. The varying responses could tell the various learning effects.
• The data could be applied to conduct replicated studies with competitive statistical models to compare the goodness-of-fit for observing the insights of education mutation amid the COVID-19 pandemic. In addition, the data can also conduct cross-regions in the greater China regions, including the Chinese Mainland, Hong Kong, and Macau. The cross-region studies could statistically extend the research generalizability if the data could be collected more out of the greater China regions. As we argued above, further studies could be conducted to compare learning outcomes with various pandemic control policies while obtaining different datasets for other countries.

## 1 Data Description

The raw data is measured from the questionnaire designed by the research team based on the literature review of the research constructs [5]. The questionnaire items are in Table 1, showing the variables' measurement scales. Q1∼Q3 variables are the second level of social media construct, which is the channel for conducting real-time online courses amid the COVID-19 pandemic. Q4 and Q5, the second level of information construct, represent the teaching and learning information that transmits in the education system. Q6 and Q7 are the same learning outcome, where Q6 questions are measured individually to apply to the structural equation modeling (SEM) technique in the related research article [1]. Q7 is a question that could be used for the other analysis technique, such as regression analysis. Q8∼Q10 variables measure the structural differentiation construct, including the second-level constructs of individual technique and instruction rule dimensions. The descriptive analysis of questionnaire items is presented in Table 1 for reference. The rest of the questions, Q11∼Q16, are the demographical measure scales with descriptive analysis results, as indicated in Table 2. The questionnaire and raw data are provided on Mendeley data at https://doi.org/10.17632/6gjyr6vm9g.1.

**Table 1:** The questionnaire and variable descriptive analysis.

Variables	Questions	Mean	St. Dev.
**Q1**	**Channel function of social media**		
	1. Social media provides information channels for knowledge transfer	4.13	0.71
	2. Social media provides information channels to answer questions.	4.05	0.84
	3. Social media provides information channels for learning activities, such as exercises.	4.03	0.86
	4. Social media provides information channels for interactions between teachers and students	4.05	0.84
	5. Social media provides information channels for group discussions	3.88	0.91
	6. Social media provides informational channels for sharing education information	4.22	0.78

**Q2**	**Educational information provided by social media**		
	1. Social media provides diverse and rich educational information.	4.02	0.84
	2. Social media provides necessary educational information.	3.95	0.85
	3. Social media provides educational information that promotes learning.	3.77	0.95
	4. Social media provides real-time educational information.	4.05	0.80
	5. Social media provides trustworthy educational information.	3.71	0.89

**Q3**	**Learning assessment functionality of social media**		
	1. Social media provides channels for submitting assignments/reports.	4.16	0.81
	2. Social media provides channels for oral presentation.	3.79	0.94
	3. Social media provides platforms for tests and exams.	3.95	0.91
	4. Social media provides functionality for auto marking.	3.67	1.01

**Q4**	**Course information sources**		
	1. Course information includes rich online links.	3.91	0.81
	2. Course information includes rich content.	3.88	0.92
	3. Course information is easy to reach.	3.97	0.90
	4. Course information is easy to retrieve/search.	3.93	0.88
	5. Course information transmit smoothly.	3.58	1.00

**Q5**	**Course content**		
	1. Course content is understandable.	3.73	0.88
	2. Course content is valuable.	3.96	0.84
	3. Course content is trustworthy.	3.88	0.84
	4. Course content has good quality.	3.91	0.81
	5. Course content is objective.	3.93	0.82
	6. Course content is secure.	3.91	0.89

**Q6**	**Overall, what level do you think about your learning output from online teaching/learning?**	3.17	1.21
	Level 1: I can memorize the learning content (retrospectively recall the course information).		
	Level 2: I can comprehend the learning content (interpretation, explanation, summarization, classification, inference, and comparison).		
	Level 3: I can apply the learning content and solve problems (using course information to perform homework, implement plans, and solve problems).		
	Level 4: I can analyze the causal relationships of learning components (I can identify the composition of curriculum content, organize the associations of content composition and knowledge systems).		
	Level 5: I can evaluate the value, judge the pros and cons, and make decisions from learning content (I can check and comment on the course content according to systematic rules and standards).		
	Level 6: I can reorganize learning content and innovate knowledge (I can develop strategies, innovate knowledge, and invent things based on learning).		

**Q7**	**Regarding the learning outcomes of the previous question, please evaluate each index individually**		
	1. Level 1: I can memorize the learning content.	4.12	0.83
	2. Level 2: I can comprehend the learning content.	4.04	0.83
	3. Level 3: I can apply the learning content and solve problems.	3.79	.088
	4. Level 4: I can analyze the causal relationships of learning content.	3.56	0.94
	5. Level 5: I can evaluate the value, judge the pros and cons, and make decisions of learning content.	3.34	1.02
	6. Level 6: I can reorganize learning content and innovate knowledge.	3.08	1.14

**Q8**	**Your personal technical ability on social media**		
	1. I have used social media for teaching/learning.	3.66	0.95
	2. My adaptability to apply social media in teaching/learning is well.	3.88	0.94
	3. I can apply social media to teach/learn.	4.03	0.83
	4. I can benefit from the social media community in teaching/learning.	3.82	0.86
	5.I can build up my knowledge system through social media.	3.67	0.90

**Q9**	**Information governance**		
	1. Information governance can improve information integration.	3.93	0.76
	2. Information governance can implement the organizational vision, plans, and structures.	3.85	0.87
	3. Information control can cooperate with the information department and functionality.	3.93	0.80
	4. Information governance reduces organizational heterogeneity.	3.67	0.89
	5. Information governance is a crucial mechanism for organizational security	3.88	0.85

**Q10**	**Teaching guides of information governance**		
	1. Information governance is better to operate from top to bottom levels.	3.82	0.83
	2. Information governance provides a blueprint for teaching/learning.	3.90	0.84
	3. Educators' knowledge and experience could form a mechanism of information governance.	3.91	0.80
	4. Information governance could promote teaching from learning.	3.90	0.86
	5. Information governance can ensure teaching/learning quality.	3.75	0.91

**Table 2:** The descriptive demographic analysis.

Variables	Questions	Frequency	Percentage
**Q11**	**Your status**		
	Teacher;	227	24.5%
	Student	699	75.5%

**Q12**	**Your gender**		
	Male;	354	38.2%
	Female	572	61.8%

**Q13**	**Your age**		
	20 or below	335	36.2%
	21-30	386	41.7%
	31-40	153	16.5%
	41-50	46	5.0%
	51-60	5	0.5%
	61 or above	1	0.1%

**Q14**	**Teaching/Learning area**		
	1. Chinese Mainland;	619	66.8%
	2. Hong Kong and Macau	307	33.2%

**Q15**	**Living area**		
	1. Chinese Mainland;	838	90.5%
	2. Hong Kong;	35	3.8%
	3. Macau;	44	4.8%
	4. Taiwan;	1	0.1%
	5. Others	8	0.9%

**Q16**	**Educational level**		
	1. High school;	21	2.3%
	2. Undergraduate;	677	73.1%
	3. Master;	179	19.3%
	4.Ph.D.	49	5.3%

## 2 Experimental Design, Materials and Methods

Table 3 shows the experimental design from the model construction to the analysis method. The research design began with the model and hypotheses construction based on the information theory intervening by the social media and structural differentiation constructs. Then a questionnaire was developed with measurement scales of variables referred to and supported by the literature. The raw data were generated through a survey that measured participants’ cognitions of the model constructs. The researchers collected a sample size of 957 participants, 926 of them were used after filtrated for valid responses. A random sampling of targeted groups was required when outsourcing the data collection to the survey company Wenjuanxing with a majority population database providing functions equivalent to Amazon Mechanical Turk [2]. The data can be referred to as the population attribute from the sample file that showed the following distributions, 24.5% were teachers, and 75.5% were students. Among them, males accounted for 38.2%, and females accounted for 61.8%. Approximately most of the respondents were younger than 30 years old. Specifically, 36.2% were under 20 years old, 41.7% were 21 to 30 years old, and 16.5% were 31 to 40 years old. The vast majority of respondents had a university education; 66.8% of the respondents' teaching/learning area was in Chinese Mainland, 33.2% were in Hong Kong SAR and Macau SAR, 90.5% lived in the Chinese Mainland, 3.8% and 4.8% live in Hong Kong SAR and Macau SAR, respectively, and only 0.1% lived in Taiwan [1]. The measurement scales meet essential reliability and validity requirements. The average variance extracted values are all above 0.5 to fit the validity criteria. Composite reliability values ranged between 0.816 and 0.899, all above 0.7. Cronbach's alpha values ranged from 0.807 to 0.897, all above 0.7. The loading index for a given construct exceeded other constructs' loadings, reflecting discriminant validity. We applied the SEM analysis method through the statistical tool Mplus to test research hypotheses corresponding to the research model.

**Table 3:** The experimental design process.

Process	Design	Criteria or Purpose
Step 1	Model and hypotheses	Literature support
Step 2	Measurement scales development	Reliability and validity tests
Step 3	Sampling strategy: Random sampling	Representative to the population
Step 4	Data collection: Wenjuanxing	Sample size
Step 5	Data curation	Valid data
Step 6	Data analysis methods: SEM	Hypotheses test and Model fitness
Step 7	Statistics tool: Mplus	Statistical flexibility

*Note:* The process details can refer to our releated research article: https://doi.org/10.1016/j.jhlste.2022.100379[1]

## Ethics Statements

All authors comply with research ethics for the data. The study is not required ethics approval because it is not human research or related settings. The data are anonymized adequately so that participants can not be identified, and informed consent was obtained at the time of original data collection through the survey company Wenjuanxing.

## CRediT authorship contribution statement

**Kang-Lin Peng:** Conceptualization, Methodology, Data curation, Formal analysis, Writing – original draft, Supervision. **Jusi Xu:** Software, Data curation, Writing – review & editing. **Xin Wang:** Data curation, Writing – review & editing. **Huawen Shen:** Resources, Data curation, Validation.

## Declaration of Competing Interest

The authors declare that they have no known competing financial interests or personal relationships that could have appeared to influence the work reported in this paper.

## Data Availability

Dataset_RTOCs (Original data) (Mendeley Data).

## Appendix Supplementary materials

Image, application 1
