# Supplementary material for: Hospitality education evolution observed in online learning dataset
Source: Data Brief. 2022 Aug 8;44:108525. doi: 10.1016/j.dib.2022.108525 (PMC9364712; doi:10.1016/j.dib.2022.108525)
Supplement: Supplementary file 1 [file mmc1.zip › DIB 108525_supplementary files/DIB 108525_Releated article_JHLSTE.pdf]

Journal of Hospitality, Leisure, Sport & Tourism Education 30 (2022) 100379

Contents lists available at ScienceDirect

Journal of Hospitality, Leisure, Sport & Tourism Education

journal homepage: www.elsevier.com/locate/jhlste

# Realtime online courses mutated amid the COVID-19 pandemic: Empirical study in hospitality program

Kang-Lin Peng[a,*], Pearl M.C. Lin[b], Jusi Xu[c], Xin Wang[a]

[a] Faculty of International Tourism and Management, City University of Macau, Macau
[b] School of Hotel and Tourism Management, The Hong Kong Polytechnic University, Hong Kong
[c] School of Hospitality and Tourism Management, Shunde Polytechnic, China

ARTICLE INFO

*Keywords:*
COVID-19 pandemic
Education mutation
Hybrid-learning
Information entropy
Real-time online courses

ABSTRACT

Real-time online courses (RTOCs), a new online learning mode, have been developed because of a longitudinal suspension of classes amid the COVID-19 pandemic worldwide. We explore an information model to review the learning process and outcomes of RTOCs, which conducted educational activities via social media. Results show that social media can be a potent mediation factor with the moderation of structural differentiation to facilitate online learning outcomes. Conclusions imply that the life-changing impact of COVID-19 has caused an evolutionary online education mode that can be hybridized with face-to-face education and massive open online courses to flourish education approaches and pedagogies.

## 1. Introduction

The blend of traditional learning and massive open online courses (MOOCs) has adaptive evolution and progressive technology development (López-Pérez et al., 2011). The evolution of education modes is smooth as expected. However, the COVID-19 pandemic has impacted the world, including education (Karalis & Raikou, 2020). The highly mutated Omicron variant has led to severe infections that governments need to delay the implementation of pandemic control strategies (Poudel et al., 2022). This study aims to argue that real-time online courses (RTOCs) are becoming a new muted educational approach, which needs echoing pedagogies in education. Starting from the spring of 2020, the pandemic caused schools to lockdown campuses and forced teachers and students to initiate online learning (Bao, 2020). Many schools require teachers and students to adopt real-time online courses (RTOCs) complemented with MOOCs during the long-term suspension of face-to-face learning activities (Peng et al., 2020). The suspension of classes without suspending learning amid the pandemic has become the prevailing phenomenon among schools (Jung et al., 2021). Schools require teachers and students to adopt online resources, such as social media, to conduct teaching and learning activities even if such platforms were not designed for education (Al Lily et al., 2020). Significant issues were encountered on the educational demand and supply sides during RTOCs in the beginning. Most teachers and students were not trained to apply social media in RTOCs (Kaplan & Haenlein, 2016). Social media functioned as an educational platform but crashed all the time because of the over-demand for capacity (Chen et al., 2020). The facilities and resources failed to handle the excess demand during the pandemic. However, RTOCs were frequently used and matured with the improvement of supply and demand capabilities when the pandemic persisted for more than one year worldwide (Brown, 1993). The pandemic has already mutated into a new education mode for teaching and will persist in the

* Corresponding author. Avenida Padre Tomás Pereira Taipa, Macau.
*E-mail address:* klpeng@cityu.mo (K.-L. Peng).

https://doi.org/10.1016/j.jhlste.2022.100379
Received 23 September 2021; Received in revised form 27 February 2022; Accepted 9 March 2022
Available online 17 March 2022
1473-8376/© 2022 Elsevier Ltd. All rights reserved.

future (Taglietti et al., 2021).

This study explores the RTOCs' learning process and outcomes. RTOCs adapt several social media platforms to transmit educational information between educators and learners. No single social media platform can offer complete functions for various subjects among schools (van Bommel et al., 2020). Teachers may require students to use Zoom for meetings (Kohnke & Moorhouse, 2020), Rain Classroom for in-class exercises and interactions (Li et al., 2021), Moodle for forum discussions (Dascalu et al., 2021), and Google Forms for quiz evaluation (Afiah & Pujiastuti, 2021). These social media help RTOCs facilitate knowledge transmission when face-to-face and MOOCs are not applicable. However, information leakage occurs because of the inconsistency of the information platforms; that is, information entropy increases to raise the chaos of the new education ecosystem between educators, students, and social media (Allen et al., 1996; McDermott, 2022). Thus, we extract the factors of information and social media with structural differentiation to explore the learning outcomes of the RTOCs-efficiency model. The objective is to observe the evolutionary mutation of RTOCs parallel with face-to-face and MOOCs learning.

## 2. Literature review

### 2.1. Evolution from traditional education, MOOCs to RTOCs

Education evolves with social and cultural changes (Baker, 2014) and technology development, especially the influence of social media (Shulla et al., 2020). Traditional education conducts face-to-face teaching and learning activities in physical schools, where teachers and students gather together for knowledge transformation (Dede, 1996). However, the school's territory also forms a barrier to prevent students who are not registered (Reich, 2020). Some top schools have abundant resources that can only be shared with upper-level students (Oakes, 1986). The uneven distribution of educational resources was allocated to different levels of students with diverse social classes, resulting in the icing-on-the-cake phenomenon of traditional education (Xing, 2013). Therefore, endeavors have been made to initiate MOOCs with online learning pedagogy since 2012 to be more oriented toward social justice (Bali et al., 2020; Gaebel, 2014). Equality opportunity in humanism is a guiding philosophy for education (Tamrat, 2020).

MOOCs emerged at the end of the 19th century and followed technology and Internet development (Kalman, 2014) to release some educational resources to break inequality. Schools' courses have opened to the public for free through the Internet, facilitating massive online courses to break through traditional education boundaries (Sandeen, 2013). Many differences have been observed between MOOCs and traditional education regarding educational cost, resources, personalized learning, interactivity, and evaluation (Daniel et al., 2015). However, pure online education cannot substitute traditional education. MOOCs are not in line with the local customization of education (Germain, 2020). Furthermore, the unequal distribution of education resources still exists because MOOCs are a supplementary educational pathway for traditional education (Lambert, 2020).

Education has evolved from face-to-face teaching, from MOOCs to hybrid pedagogies (Defaweux et al., 2019). However, the COVID-19 outbreak suddenly suspended the traditional approach to control and prevent the pandemic. MOOCs were unable to become an alternative because of the pedagogy's nature and the lack of interaction in real-time interaction (Sinclair & Kalvala, 2016). RTOCs through social media are recommended during schools' shutdowns because of teaching and learning obligations, better time management, and similar teaching and learning arrangements. However, RTOCs had problems when it was first implemented. For example, teachers and students could not run RTOCs smoothly because of the incapability to use social media for teaching and learning (Jogezai et al., 2021). On the supply side, social media cannot quickly handle the vast volume of excess demand. After a long-term accommodation, both sides of RTOCs started evolving as a regular educational mode, from quantitative to qualitative changes (Jacobson et al., 2019). The demand side has gained substantial improvements in the capability of RTOCs, and the supply side has invested strategic resources to attract educational users. Social media investments are expected to continue, such as the regeneration and use of Olympic investments after the games (Gold & Gold, 2020). A new approach, RTOCs, is added to multiple paths like traditional education and MOOCs to form the hybrid education system (Nguyen, 2015).

### 2.2. Use social media for RTOCs

#### 2.2.1. Information system and learning outcome

Education is a typical information transmitting and processing system, where teachers deliver information to students through pedagogies (Loughran, 2013). Despite the recent growth of tutorship and mentorship to stimulate students' self-learning (Kirillova & Au, 2020), most teaching activities have mainly adopted the transmission-reception mode to deliver information to students (Z.-g. Wang, 2007). These teaching modes dovetail with two main research streams in how information influences learning outcomes (Hosier, 2017). The first stream focuses on teaching (Barber & Mourshed, 2007; Darling, 2000; Yin et al., 2016). Fauth et al. (2014) proposed three dimensions of teaching quality, namely, classroom management, cognitive activation, and supportive climate, suggesting that teachers should maintain a quiet learning environment, stimulate students' critical thinking, and provide continuous support to help information transmission.

The second research stream focuses on learning how information learned by students helps achieve better learning outcomes, including six levels of Bloom's taxonomy of cognitive development (Abbasi & Kazi, 2014; Bloom et al., 1984). Students obtain information from teachers, teaching materials, and facilities (Z. Wang, 2007). Many schools invest in various information resources to help students digest information (Serenko et al., 2012). The effectiveness of these information resources relies on students' learning desires and how these information resources are organized for students to choose, evaluate, and use. Based on these two perspectives, this contention stems from behavioral theories that highlight that teachers should transmit the knowledge well-organized (L. Wang,

2007) and students should learn with resources. Thus, many studies have adopted information literacy in the online learning environment to design an educational information system that assists teachers and students in processing and handling learning outcomes (Detlor et al., 2011; Samson, 2010).

**H1**. Information positively influences learning outcomes.

### 2.2.2. *Information and social media*

The educational information system has evolved from traditional face-to-face education with the supplement of MOOCs via information platforms (Bralić & Divjak, 2018). The RTOCs via social media are chosen as an alternative mode when suspending classes during the pandemic when face-to-face activities and MOOCs are not applied (Ali, 2020). We stated that social media had improved their facilities and capacities to meet the requirements of RTOCs (Afiah & Pujiastuti, 2021; Dascalu et al., 2021; Gold & Gold, 2020; Li et al., 2021). A research gap in investigating how RTOCs were utilized to build an effective information system can help students achieve better learning outcomes (Azevedo et al., 2021).

**H2**. Information positively influences social media.

### 2.2.3. *Social media and learning outcome*

The sudden education mutation, RTOCs conducted via social media, introduces deviations to the existing educational information system. Many institutions have utilized RTOCs through various social media platforms, such as Zoom, MS Teams, and WeChat RC, which are not tailormade for teaching purposes (Afiah & Pujiastuti, 2021; Dascalu et al., 2021; Feng et al., 2019; Li et al., 2021). These platforms bring many uncontrollable factors influencing students' learning outcomes (Scherer et al., 2019), for example, technical issues, infeasible monitoring of students' learning progress, and test cheating (Rasheed et al., 2020). However, the long-term high-frequency adoption of RTOCs amid the pandemic has changed from quantitative to qualitative improvement of learning outcomes (Azevedo et al., 2021).

**H3**. Social media positively influences learning outcomes.

### 2.2.4. *The mediation effect of social media*

COVID-19 persists in causing RTOCs continuous somewhere with severe confirmed cases. Large-scale national resources have been invested in supporting remote learning during the COVID-19 pandemic period (Ali, 2020). RTOCs were taught and learned through social media and have become mainstream educational activities because of the failed adoption of face-to-face and MOOCs learning (Kumar & Vulichi, 2021). Teachers and students have adopted RTOCs via social media for knowledge transmission to achieve learning outcomes (Linder, 2021). For example, students joined social media for online learning, teachers turned to social media to conduct online courses, and assessments could be conducted online (Rasheed et al., 2020). Finally, these teaching and learning activities were quickly adopted because social media evolved with the education requirements (Reimers, 2022).

**H4**. Social media plays a mediating role between information and learning outcomes.

## 2.3. *Structural differentiation in educational information system*

Shannon (1948) pioneered developing a mathematical model called information entropy using probability theory to calculate information quantity based on the information volume transmitted within a unit of time. This model stems from studies of uncertainty to eliminate information chaos that cause the information to sink in the information source channel (Wang & Li, 2003). It has widely been applied in different study fields, such as information system (Liang et al., 2006), medicine (Tsai et al., 2008), and manufacturing (Jung et al., 2011), to evaluate the information delivery process in influencing people's behavioral and physiological responses (Jing et al., 2018). Although education is an extensive information system, it has received limited academic attention on eliminating information entropy. Few studies include Z. Wang (2007), who considered four interventions to determine information entropy at the end of the teaching process: teachers' knowledge, teaching methods, teaching experience, and expression ability, defined as teachers' individual capability. These four factors focus on the teacher's capability in the teaching process (Lei & So, 2021; Marfuah et al., 2022). In addition, Sheng et al. (2017) focused on the teaching level assessment. Other factors were discussed on the structural differentiation of educational information systems. Qu et al. (2015) advanced it by incorporating other class management interventions, for example, the flexibility of teaching activities and the proportion of teaching time to determine information transmission quality and predict information entropy. We defined interventions from institution rules. The literature has also viewed the application of information technology in learning activities in an optimistic way, suggesting that information technology can help students achieve better learning outcomes through improving information technology capability (Sugandi & Kurniawan, 2018). For example, Rusmono et al. (2019) adopted an experimental approach to demonstrate that mobile teaching resulted in a 16.8% increase in students' learning outcomes. The pandemic has forced many institutions to utilize various social media platforms for RTOCs with their institution rules and training teachers and students' technology capabilities to reduce educational information entropy.

Educational mutation happens when the capability is enhanced on the demand side and resources are invested on the supply side. RTOCs will not disappear after the pandemic because educational mutation has become irreversible between stakeholders' intangible capability and tangible investment in RTOCs. A pedagogy that can meet the characteristics of RTOCs is also necessary to develop the multi-pathway with traditional education and MOOCs.

**H5.** Structural differentiation plays a moderating role between social media and learning outcomes.

## 3. Methodology

### 3.1. Research model

Information influences knowledge integration and learning outcomes during knowledge exchange because of enhanced activation (Erkens & Bodemer, 2019). When traditional face-to-face teaching and learning activities were suspended amid the pandemic, social media platforms, such as the video-conferencing platform Zoom, were widely adopted as the pathway for RTOCs (Ali, 2020; Kohnke & Moorhouse, 2020). However, social media was not tailor-made to educational purposes that influence the learning outcome's effectiveness (Feng et al., 2019). The moderation of structural differentiation is necessary to resume teaching and learning systems (Scherer et al., 2019). Social media can then be a reliable mediation channel between educational information and learning outcomes as a new pathway of knowledge transformation amid or after the pandemic (Lee et al., 2019). The moderated mediation educational model of RTOCs through social media is constructed in Fig. 1.

### 3.2. Research design

The RTOCs measurement tool consisted of four constructs: information, learning outcome, social media, and structural differentiation. The eleven items in information focus on measuring information accessibility and usability (Lee et al., 2002). Learning outcome consists of two dimensions, essential ability and creative ability, with six items of learning outcome (Chauhan, 2014). Social media consists of eleven channel functionality items and four function items. Social media measures the degree of providing the educational content in the teaching and learning activities (Bertot et al., 2006; Tandon et al., 2016, 2018). The structural differentiation of social media measures institutional rules (Chen et al., 2015; Hina et al., 2019) and individual capability (Evans et al., 2014) (Appendix 1).

The study is conducted in the Chinese Mainland, Hong Kong SAR, and Macau SAR. Data were collected by recruiting 956 respondents; 926 responses were adopted after the valid screening through a cooperative survey company. A random sampling of targeted groups was required when outsourcing the data collection to the survey company Wenjuanxing, a platform with a majority of population database providing functions equivalent to Amazon Mechanical Turk. We asked the company to deliver the designed questionnaire to teachers and students in hospitality programs.

Reliability and validity of all constructs showed that the questionnaire is proper for measurement (Appendix 2). Data analysis applied the structural equation model with Mplus to examine the CFA model and research hypothesis (Byrne, 2013).

## 4. Results and discussions

### 4.1. Profile of respondents

Among the survey respondents, 24.5% were teachers, and 75.5% were students. Among them, males accounted for 38.2%, and females accounted for 61.8%. Approximately most of the respondents were younger than 30 years old. Specifically, 36.2% were under 20 years old, 41.7% were 21–30 years old, and 16.5% were 31–40 years old. The vast majority of respondents had a university education; 66.8% of the respondents' teaching/learning area was in Chinese Mainland, 33.2% were in Hong Kong SAR and Macau SAR, 90.5% lived in Chinese Mainland, 3.8% and 4.8% live in Hong Kong SAR and Macau SAR, respectively, and only 0.1% lived in Taiwan. Table 1 shows the demographic of the survey sample.

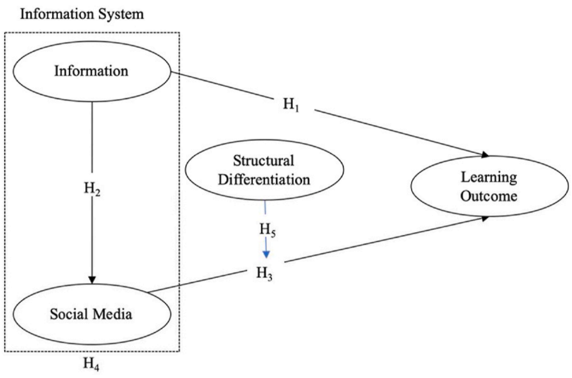


**Fig. 1.** The intervention model of RTOCs.

**Table 1**
Descriptive statistics of respondents' characteristics.

| Items | Classification | Frequency | Percentage (%) |
|---|---|---|---|
| Identity | Teacher | 227 | 24.5 |
| | Student | 699 | 75.5 |
| Gender | Male | 354 | 38.2 |
| | Female | 572 | 61.8 |
| Age | 20 or below | 335 | 36.2 |
| | 21–30 | 386 | 41.7 |
| | 31–40 | 153 | 16.5 |
| | 41–50 | 46 | 5 |
| | 51–60 | 5 | 0.5 |
| | 61 or above | 1 | 0.1 |
| Education | High school | 21 | 2.3 |
| | Bachelor's degree | 677 | 73.1 |
| | Master's degree | 179 | 19.3 |
| | Doctor's degree | 49 | 5.3 |
| Current living area | Chinese Mainland | 838 | 90.5 |
| | Hong Kong SAR | 35 | 3.8 |
| | Macau SAR | 44 | 4.8 |
| | Taiwan | 1 | 0.1 |
| | Other | 8 | 0.9 |
| Teaching/learning area | Chinese Mainland | 619 | 66.8 |
| | Hong Kong SAR and Macau SAR | 307 | 33.2 |

### 4.2. The measurement model

Reliability and validity values for constructs all meet the measurement requirements. The average variance extracted values are all above 0.5 to fit the validity criteria. Composite reliability values ranged between 0.816 and 0.899, all above 0.7. Cronbach's alpha values ranged from 0.807 to 0.897, all above 0.7 (Appendix 2). The standardized results obtained from the structural model, after 1000 bootstrap iterative calculations in Mplus, show that the data fit the model well: $\chi^2$(df) = 2.95, RMSEA = 0.046, CFI = 0.914, TLI = 0.908, SRMR = 0.041. The loading index for a given construct exceeded that of other constructs' loadings, reflecting discriminant validity, as shown in Table 2.

### 4.3. Mediation analyses of social media

We tested the mediation effect of 1000 bootstrap samples by Mplus and found that social media was significantly related to information ($\beta = 0.919$, $p < 0.001$) and learning outcomes ($\beta = 0.718$, $p < 0.001$). However, after the mediation effect of social media, the relationship between information and learning outcome is insignificant ($\beta = 0.201$, $p > 0.5$). The direct effect of the mediating effect between information and learning outcome was insignificant, which indicated that social media plays a full mediator role between information and learning outcome. Therefore, $H_4$ is supported. Table 3 shows the results of the mediation model.

### 4.4. Moderation analyses of structural differentiation

Fig. 1 demonstrated that structural differentiation has a moderation effect between social media and learning outcomes. The standardized coefficient from "social media*structural differentiation" to learning outcome was −0.056 ($p < 0.05$), which illustrated that the moderation effect was statistically significant. Furthermore, we picked low and high moderator values for structural differentiation and added or subtracted standard deviation below the mean value of moderating variable (structural differentiation: 3.86 ± 0.84). Then, we conducted path analysis in Mplus. Fig. 3 intuitively provides evidence of the moderating effect of structural differentiation on social media and learning outcomes. The statistically significant interaction between social media and learning outcome shows that the positive correlation between social media and learning outcome is robust at a lower level of structural differentiation. In comparison, at a higher level of structural differentiation, the positive correlation between social media and learning outcomes is weaker.

Consequently, the moderating variable of structural differentiation exerted a negative moderating effect on the second stage of the indirect effect between social media and learning outcome. Thus, structural differentiation played a moderating role in social media

**Table 2**
Discriminant validity.

| | IN | LO | SM | SD |
|---|---|---|---|---|
| Information (IN) | 1 | | | |
| Learning outcome (LO) | 0.759 | 1 | | |
| Social media (SM) | 0.866 | 0.752 | 1 | |
| Structural differentiation (SD) | 0.859 | 0.774 | 0.870 | 1 |

**Table 3**
Standardized direct, indirect, and total effects.

| | Estimate | Product of coefficient | | 95% Confidence interval (1000 bootstrap samples) | |
|---|---|---|---|---|---|
| | | S.E. | Est./S.E. | Lower | Upper |
| Total Effect | 0.919*** | 0.036 | 25.814 | 0.849 | 0.993 |
| Indirect Effect | 0.718*** | 0.177 | 4.064 | 0.452 | 1.160 |
| Direct Effect | 0.201 | 0.186 | 1.079 | −0.254 | 0.508 |

*Note.* Values represent standardized regression coefficients. ***$p < 0.001$.

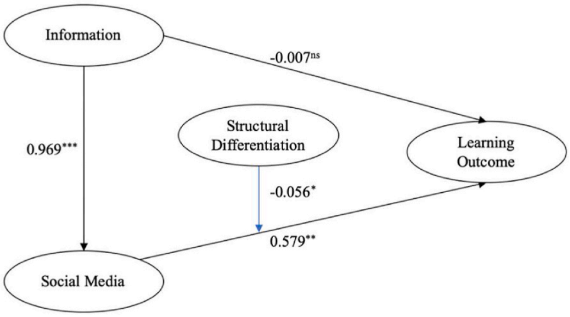


**Fig. 2.** The testing model.
*Note.* Values represent standardized regression coefficients. *$p < 0.05$; **$p < 0.01$; ***$p < 0.001$.

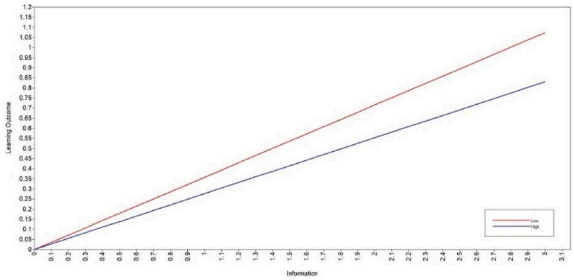


**Fig. 3.** Relationship between information and learning outcome at different levels of structural differentiation.

and learning outcomes. $H_5$ was supported accordingly.

Based on the statistical findings, the five inter-construct relationships were re-summarized in Table 4, together with the empirical results of supported $H_1$–$H_5$.

## 4.5. Discussions

### 4.5.1. Model interpretation

Fig. 2 and Table 4 show that the structural model of RTOCs is sustained. The results prove that educational information systems, including social media, can offer intervention functions to maintain learning outcomes. Hypothesis 1 is significant with a negative path coefficient that tells the advert effect of learning from chaotic information, especially when RTOCs were first introduced. Teachers and

**Table 4**
Standardized path coefficients for structural mode.

| Hypothesis | Standardized path coefficient | Testing result |
|---|---|---|
| $H_1$: IN→LO | −0.007$^{ns}$ | Not Supported |
| $H_2$: IN→SM | 0.969*** | Supported |
| $H_3$: SM→LO | 0.579** | Supported |
| $H_4$: IN→SM→LO | 0.718*** | Supported |
| $H_5$: SD on SM→LO (moderation effect) | −0.056* | Supported |

*Note.* Values represent standardized regression coefficients. *$p < 0.05$; ***$p < 0.001$, ns: insignificant.

learners should adopt proper social media to conduct educational activities when classes are suspended because of the prevention and control of the pandemic. This action also accords with earlier observations, which showed that information helps achieve better learning outcomes (Abbasi & Kazi, 2014; Wang, 2007), especially during a pandemic. However, social media's direct ($H_2$) and indirect effects ($H_4$) have converted the learning effect into positive outcomes that show the remedy measures from technology. Although the beginning stage of RTOCs is orderless, later social media helps teachers and learners interact, get accustomed to the online learning environment, and improve learning outcomes. This study supports evidence from previous observations (e.g. Linder, 2021; Bralić & Divjak, 2018). It shows that the RTOC mode is a helpful alternative when face-to-face educational activities are suspended and MOOCs are not suitable. It also further supports the idea of Ali (2020) that the RTOCs via social media are chosen as an alternative mode. The moderation effect of the structural differentiation of institutional rules and individual capabilities in social media can also reduce the information entropy to raise the learning effects ($H_5$) (Matei & Britt, 2017). The result expands the understanding of structural differentiation on the basics of previous studies of Bo (2019). Educational authorities could lead the choice of social media, suggest few media that most teachers and students accept, and train teachers and learners to improve their technical ability to use social media for teaching activities. These measures could help reduce the chaos and uncertainty of the information system used to conduct teaching and learning activities for learning outcomes.

#### 4.5.2. *Adoptive pedagogy for the evolution of the RTOCs*

The sudden emergence of RTOCs in China, Hong Kong SAR, and Macau SAR has evolved into an evolved educational approach amid the pandemic. Teachers and students on the demand side have strengthened their online teaching and learning abilities. Social media platforms on the supply side have also up-scaled their hardware and software facilities. A new RTOCs ecosystem evolved to a third education approach beyond traditional face-to-face courses and MOOCs in China, Hong Kong SAR, and Macau SAR. The RTOC model has become a robust education system that needs echoing pedagogies to confront the fluctuating circumstances of the pandemic. Knowledge transformation parallels traditional mode and MOOCs because of the prolonged pandemic from 2019 to 2022. The RTOCs are not concerned about what kinds of social media platforms have been applied. The most crucial issue is to achieve students' learning outcomes, which are provable education content (Kennedy, 2006), transformative knowledge, empathy analysis, and action capabilities (Munroe & Pearson, 2006). RTOCs help the knowledge conversion process achieve learning outcomes into the cognitive, skill, and attitude capabilities (Halawi et al., 2009). Nevertheless, the RTOC intervention model is necessary to form its adaptive pedagogy that can guarantee the qualifying role of education on the evolving pathway.

## 5. Conclusions

### 5.1. *Conclusion*

The education evolution moves from traditional face-to-face to MOOCs modes smoothly and advanced information technology until the end of outbreaks and prolonged pandemic circumstances. The pandemic has led to a sudden muted RTOC mode when teaching and learning happen despite restricted campus access, similar to the condition in the Greater China regions. Educational information transmission to learning outcomes could be chaotic without the intervention of information systems and institutional governance during the pandemic. While the pandemic variant Omicron is still raging, the RTOCs have become a multi-channel of teaching and learning after a long-term adaptation of educational information supply and demand requirements. An echoing pedagogy is needed for the mutated educational mode implemented with the traditional face-to-face and MOOCs approaches.

### 5.2. *Research implication*

The practical implication of this study is that the continuous COVID-19 variants have sustained RTOCs, which have become a new teaching and learning mode that complement face-to-face and MOOCs approaches during the changing conditions brought by the pandemic. The mixed-mode has been applied to educational activities and the fluctuating circumstances of the control and prevention measures of the pandemic. The policy of class suspension without suspending learning amid the pandemic via RTOCs has prevailed for areas with severe confirmed cases. After adopting remote teaching and learning through social media for educational activities during a period, teachers and students were trained to use RTOCs for students' learning outcomes. Social media has dramatically improved facilities, equipment, bandwidth, and network speed to meet educational requirements on the technology supply side. The intervention of institution governance was also exercised effectively in the new educational ecosystem. The RTOC mode has evolved from quantitative adoption to quality improvement to parallel development with face-to-face education and MOOCs. Even though the pandemic has negatively influenced our education system, educators and students have found their way out for continuous learning.

### 5.3. *Recommendation*

This study recommends getting used to the multi-channel teaching and learning approaches following the tight or relaxed pandemic prevention measures caused by COVID-19 variants. The RTOCs have been parallel to the face-to-face approach for some students who may be quarantined because of the special pandemic conditions. Thus, institutions need to design an educational media platform suitable for RTOCs since many regions have prolonged control and prevention measures. The RTOC mode has evolved to be a parallel educational approach with traditional face-to-face education and MOOCs at different times and places in need. The hybrid online and offline parallel educational approaches should also come out with mixed pedagogies. The pandemic's influence persists

even though it caused the education system's initial disorder. However, the mutated educational ecosystem for continuous knowledge conversion and transmission may be suitable for education systems to accumulate experiences to deal with similar situations in the future.

## Author statement

**Kang-Lin PENG**: Conceptualization, Methodology, Formal analysis, Model construction, Writing- Original draft preparation, Supervision. **Pearl M.C. LIN**: Resources, Data curation, Writing - Review & Editing, Validation. **Jusi Xu**: Software, Writing- Reviewing and Editing. **Xin Wang**: Writing - Review & Editing.

## Appendix 1. Variable measurement

| Constructs | Dimensions | Items | Reference |
|---|---|---|---|
| Information | | Course information has rich online links. | |
| | | Course information has good content. | |
| | | Course information is easy to obtain. | |
| | | Course information is easy to retrieve/search. | |
| | | Course information is delivered without hindrance. | |
| | | Course information is easy to understand. | |
| | | Course information is valuable. | |
| | | Course information is trustworthy. | |
| | | Course information has high quality. | |
| | | Course information is objective. | |
| | | Course information is safe. | |
| Learning outcome | Basic ability | Level 1: Able to remember learning content | |
| | | Level 2: Able to understand the learning content | |
| | | Level 3: Able to apply learning content and solve problems | |
| | Creative ability | Level 4: Able to analyze the details of learning content or causality | |
| | | Level 5: Able to evaluate the value of learning content, judge the pros and cons, and make decisions | |
| | | Level 6: Able to reorganize, create, and innovate knowledge | |
| Social media | Channel | Social media provides an information channel for knowledge transfer. | |
| | | Social media provides information channels to resolve confusion. | |
| | | Social media provides information channels for learning activities, such as inter-class activities and homework exercises. | |
| | | Social media provides an information channel for teacher-student interaction. | |
| | | Social media provides an information channel for group discussions. | |
| | | Social media provides information channels for sharing information. | |
| | | Social media provides diverse and rich educational information. | |
| | | Social media provides educational information needed for learning. | |
| | | Social media provides educational information that promotes learning. | |
| | | Social media provides real-time updated educational information. | |
| | | Social media provides a channel to submit assignments/reports. | |
| | Function | Social media provides trusted educational information. | |
| | | Social media provides a platform for oral reporting. | |
| | | Social media provides a platform for subject examinations. | |
| | | Social media provides the function of automatic scrolling. | |
| Structural differentiation | | Information control can improve the degree of information integration. | |
| | | Information control can implement organizational vision, plans and structure. | |
| | | Information control can cooperate with information management organization and functions. | |
| | | Information control can reduce organizational heterogeneity. | |
| | | Information control is a key mechanism for organizational security. | |
| | | The information control mechanism should be developed from top to bottom (from the top of the organization to the grassroots). | |
| | | Information control provides a blueprint for teaching/learning development. | |
| | | The knowledge and experience of educators will form an information control mechanism. | |
| | | Information control can guide teaching and learning. | |
| | | Information control can ensure teaching/learning quality. | |

## Appendix 2. Reliability and validity of all constructs

| Constructs | Items | Factor loadings | CR | AVE | Cronbach's α |
|---|---|---|---|---|---|
| Information | IN1 | 0.682 | 0.899 | 0.547 | 0.897 |

(*continued on next page*)

(*continued*)

| Constructs | Items | Factor loadings | CR | AVE | Cronbach's α |
|---|---|---|---|---|---|
| | IN2 | 0.659 | | | |
| | IN3 | 0.651 | | | |
| | IN4 | 0.648 | | | |
| | IN5 | 0.627 | | | |
| | IN6 | 0.670 | | | |
| | IN7 | 0.708 | | | |
| | IN8 | 0.666 | | | |
| | IN9 | 0.716 | | | |
| | IN10 | 0.684 | | | |
| | IN11 | 0.637 | | | |
| Learning outcome | BAS1 | 0.597 | 0.816 | 0.528 | 0.807 |
| | BAS2 | 0.670 | | | |
| | BAS3 | 0.754 | | | |
| | CRE1 | 0.718 | | | |
| | CRE2 | 0.621 | | | |
| | CRE3 | 0.538 | | | |
| Social media | CHAN1 | 0.660 | 0.899 | 0.572 | 0.895 |
| | CHAN2 | 0.624 | | | |
| | CHAN3 | 0.597 | | | |
| | CHAN4 | 0.588 | | | |
| | CHAN5 | 0.568 | | | |
| | CHAN6 | 0.630 | | | |
| | CHAN7 | 0.674 | | | |
| | CHAN8 | 0.683 | | | |
| | CHAN9 | 0.646 | | | |
| | CHAN10 | 0.624 | | | |
| | CHAN11 | 0.619 | | | |
| | FUNC1 | 0.580 | | | |
| | FUNC2 | 0.554 | | | |
| | FUNC3 | 0.593 | | | |
| | FUNC4 | 0.488 | | | |
| Structural Differentiation | SD1 | 0.749 | 0.897 | 0.566 | 0.895 |
| | SD2 | 0.690 | | | |
| | SD3 | 0.707 | | | |
| | SD4 | 0.607 | | | |
| | SD5 | 0.678 | | | |
| | SD6 | 0.612 | | | |
| | SD7 | 0.709 | | | |
| | SD8 | 0.716 | | | |
| | SD9 | 0.687 | | | |
| | SD10 | 0.655 | | | |

## References


Abbasi, S., & Kazi, H. (2014). Measuring effectiveness of learning chatbot systems on Student's learning outcome and memory retention. *Asian Journal of Applied Science and Engineering, 3*(2), 251–260.

Afiah, N. R., & Pujiastuti, H. (2021). Developing A learning media on limit of algebraic functions by using Google forms. *Journal of Medives: Journal of Mathematics Education IKIP Veteran Semarang, 5*(2), 353–362. https://doi.org/10.31331/medivesveteran.v5i2.1732

Al Lily, A. E., Ismail, A. F., Abunasser, F. M., & Alqahtani, R. H. A. (2020). Distance education as a response to pandemics: Coronavirus and Arab culture. *Technology in Society, 63*, Article 101317.

Ali, W. (2020). Online and remote learning in higher education institutes: A necessity in light of COVID-19 pandemic. *Higher Education, 10*(3), 16–25.

Allen, B. S., Otto, R. G., & Hoffman, B. (1996). Media as lived environments: The ecological psychology of educational technology. *Handbook of research for educational communications and technology, 2*, 215–241. https://doi.org/10.4324/9781410609519

Azevedo, J. P., Hasan, A., Goldemberg, D., Geven, K., & Iqbal, S. A. (2021). Simulating the potential impacts of COVID-19 school closures on schooling and learning outcomes: A set of global estimates. *The World Bank Research Observer, 36*(1), 1–40. https://10.1093/wbro/lkab003.

Baker, D. (2014). *The schooled society: The educational transformation of global culture*. Stanford University Press.

Bali, M., Cronin, C., & Jhangiani, R. S. (2020). Framing open educational practices from a social justice perspective. *Journal of Interactive Media in Education, 2020*(1).

Bao, W. (2020). COVID-19 and online teaching in higher education: A case study of peking university. *Human Behavior and Emerging Technologies, 2*(2), 113–115.

Barber, M., & Mourshed, M. (2007). *How the world's best-performing schools systems come out on top*. McKinsey & Company.

Bertot, J. C., Snead, J. T., Jaeger, P. T., & McClure, C. R. (2006). Functionality, usability, and accessibility: Iterative user-centered evaluation strategies for digital libraries. *Performance Measurement and Metrics, 7*(1), 17–28. https://doi.org/10.1108/14678040610654828

Bloom, B. S., Krathwohl, D. R., & Masia, B. B. (1984). Bloom taxonomy of educational objectives. In *Allyn and Bacon*. Pearson Education.

van Bommel, J., Randahl, A.-C., Liljekvist, Y., & Ruthven, K. (2020). Tracing teachers' transformation of knowledge in social media. *Teaching and Teacher Education, 87*, Article 102958.

Bo, L. (2019). National governance capacity building under the perspective of structural differentiation. *Teaching and Research, 53*(2), 104.

Bralić, A., & Divjak, B. (2018). Integrating MOOCs in traditionally taught courses: Achieving learning outcomes with blended learning. *International Journal of Educational Technology in Higher Education, 15*(1), 1–16. https://doi.org/10.1186/s41239-017-0085-7

Brown, N. (1993). Art education and the mutation of art. *Visual Arts Research*, 63–84.

Byrne, B. M. (2013). *Structural equation modeling with Mplus: Basic concepts, applications, and programming*. routledge. https://doi.org/10.4324/9780203807644

Chauhan, A. (2014). Massive open online courses (MOOCS): Emerging trends in assessment and accreditation. *Digital Education Review*, (25), 7–17.
Chen, Y., Ramamurthy, K., & Wen, K.-W. (2015). Impacts of comprehensive information security programs on information security culture. *Journal of Computer Information Systems, 55*(3), 11–19.
Chen, X., Zou, D., Cheng, G., & Xie, H. (2020). *Detecting latent topics and trends in educational technologies over four decades using structural topic modeling: A retrospective of all volumes of computer & education*. Computers & Education, Article 103855.
Daniel, J., Cano, E. V., & Cervera, M. G. (2015). The future of MOOCs: Adaptive learning or business model? *International Journal of Educational Technology in Higher Education, 12*(1), 64–73.
Darling, H. (2000). Teacher quality and student achievement: A review of state policy evidence. *Education Policy Analysis Archives, 8*(1), 1–44.
Dascalu, M.-D., Ruseti, S., Dascalu, M., McNamara, D. S., Carabas, M., Rebedea, T., & Trausan-Matu, S. (2021). Before and during COVID-19: A cohesion network analysis of students' online participation in moodle courses. *Computers in Human Behavior, 121*, Article 106780. https://doi.org/10.1016/j.chb.2021.106780
Dede, C. (1996). The evolution of distance education: Emerging technologies and distributed learning. *American Journal of Distance Education, 10*(2), 4–36. https://doi.org/10.1080/08923649609526919
Defaweux, V., Delbrassine, D., Dozo, B.-O., Etienne, A.-M., Centi, V., D'Anna, V., Multon, S., Pesesse, L., Stassart, C., & Van de Poël, J.-F. (2019). To combine a MOOC to a regular face-to-face course–A study of three blended pedagogical patterns. *Proceedings of Work in Progress Papers of the Research, Experience and Business Tracks at EMOOCs*, 210–217, 2019 http://hdl.handle.net/2268/235271.
Detlor, B., Julien, H., Willson, R., Serenko, A., & Lavallee, M. (2011). Learning outcomes of information literacy instruction at business schools. *Journal of the American Society for Information Science and Technology, 62*(3), 572–585.
Erkens, M., & Bodemer, D. (2019). Improving collaborative learning: Guiding knowledge exchange through the provision of information about learning partners and learning contents. *Computers & Education, 128*, 452–472.
Evans, C., Hackney, R., Rauniar, R., Rawski, G., Yang, J., & Johnson, B. (2014). Technology acceptance model (TAM) and social media usage: An empirical study on facebook. *Journal of Enterprise Information Management, 27*(1), 6–30. https://doi.org/10.1108/JEIM-04-2012-0011
Fauth, B., Decristan, J., Rieser, S., Klieme, E., & Büttner, G. (2014). Student ratings of teaching quality in primary school: Dimensions and prediction of student outcomes. *Learning and Instruction, 29*, 1–9.
Feng, S., Wong, Y. K., Wong, L. Y., & Hossain, L. (2019). The internet and Facebook usage on academic distraction of college students. *Computers & Education, 134*, 41–49.
Gaebel, M. (2014). *MOOCs: Massive open online courses* (Vol. 11). EUA Geneva.
Germain, M.-L. (2020). Web and education as disruptors of traditional education and development of future students and workers. In *Disruptive and emerging technology trends across education and the workplace* (pp. 170–192). IGI Global.
Gold, J. R., & Gold, M. M. (2020). *Land remediation, event spaces and the pursuit of Olympic legacy*. Geography Compass, Article e12495.
Halawi, L. A., McCarthy, R. V., & Pires, S. (2009). An evaluation of e-learning on the basis of Bloom's taxonomy: An exploratory study. *The Journal of Education for Business, 84*(6), 374–380.
Hina, S., Selvam, D. D. D. P., & Lowry, P. B. (2019). Institutional governance and protection motivation: Theoretical insights into shaping employees' security compliance behavior in higher education institutions in the developing world. *Computers & Security, 87*, Article 101594.
Hosier, A. (2017). Creating learning outcomes from threshold concepts for information literacy instruction. *College & Undergraduate Libraries, 24*(1), 1–13.
Jacobson, M. J., Levin, J. A., & Kapur, M. (2019). Education as a complex system: Conceptual and methodological implications. *Educational Researcher, 48*(2), 112–119. https://doi.org/10.3102/0013189X19826958
Jing, S., Li, B., & Yao, R. (2018). Exploring the "black box" of thermal adaptation using information entropy. *Building and Environment, 146*, 166–176.
Jogezai, N. A., Baloch, F. A., Jaffar, M., Shah, T., Khilji, G. K., & Bashir, S. (2021). Teachers' attitudes towards social media (SM) use in online learning amid the COVID-19 pandemic: The effects of SM use by teachers and religious scholars during physical distancing. *Heliyon, 7*(4), Article e06781.
Jung, J.-Y., Chin, C.-H., & Cardoso, J. (2011). An entropy-based uncertainty measure of process models. *Information Processing Letters, 111*(3), 135–141.
Jung, J., Horta, H., & Postiglione, G. A. (2021). Living in uncertainty: The COVID-19 pandemic and higher education in Hong Kong. *Studies in Higher Education, 46*(1), 107–120. https://doi.org/10.1080/03075079.2020.1859685
Kalman, Y. M. (2014). A race to the bottom: MOOCs and higher education business models. *Open Learning: The Journal of Open, Distance and e-Learning, 29*(1), 5–14.
Kaplan, A. M., & Haenlein, M. (2016). Higher education and the digital revolution: About MOOCs, SPOCs, social media, and the Cookie Monster. *Business Horizons, 59*(4), 441–450.
Karalis, T., & Raikou, N. (2020). Teaching at the times of COVID-19: Inferences and implications for higher education pedagogy. *International Journal of Academic Research in Business and Social Sciences, 10*(5), 479–493. https://doi.org/10.6007/IJARBSS/v10-i5/7219
Kennedy, D. (2006). *Writing and using learning outcomes: A practical guide*. University College Cork.
Kirillova, K., & Au, W. C. (2020). How do tourism and hospitality students find the path to research? *Journal of Teaching in Travel & Tourism, 20*(4), 284–307.
Kohnke, L., & Moorhouse, B. L. (2020). Facilitating synchronous online language learning through Zoom. *RELC Journal.* , Article 0033688220937235. https://doi.org/10.1177/0033688220937235
Kumar, R., & Vulichi, S. R. (2021). Post covid-19: How it might cause change and impact the education in India. *ASEAN Journal of Basic and Higher Education, 5*, 65–74. https://paressu.org/online/index.php/aseanjbh/article/view/289.
Lambert, S. R. (2020). Do MOOCs contribute to student equity and social inclusion? A systematic review 2014–18. *Computers & Education, 145*, Article 103693.
Lee, C.-j., Kim, K., & Kang, B.-A. (2019). A moderated mediation model of the relationship between media, social capital, and cancer knowledge. *Health Communication, 34*(6), 577–588.
Lee, Y. W., Strong, D. M., Kahn, B. K., & Wang, R. Y. (2002). Aimq: A methodology for information quality assessment. *Information Management, 40*(2), 133–146.
Lei, S. I., & So, A. S. I. (2021). Online teaching and learning experiences during the COVID-19 pandemic–A comparison of teacher and student perceptions. *Journal of Hospitality and Tourism Education, 33*(3), 148–162. https://doi.org/10.1080/10963758.2021.1907196
Liang, J., Shi, Z., Li, D., & Wierman, M. J. (2006). Information entropy, rough entropy and knowledge granulation in incomplete information systems. *International Journal of General Systems, 35*(6), 641–654.
Linder, T. (2021). *Intelligence-captivated policing: Real-time operations centres and real-time situational awareness in Canadian police services*.
Li, Q., Wu, Y., Jiang, H., Huang, W., & Xuan, A. (2021). The application of "Rain class" in the teaching of systematic anatomy of undergraduate students. 2021 2nd asia-pacific conference on image processing. *Electronics and Computers*, 658–661. https://doi.org/10.1145/3452446.3452606
López-Pérez, M. V., Pérez-López, M. C., & Rodríguez-Ariza, L. (2011). Blended learning in higher education: Students' perceptions and their relation to outcomes. *Computers & Education, 56*(3), 818–826.
Loughran, J. (2013). *Developing a pedagogy of teacher education: Understanding teaching & learning about teaching.* Routledge. https://doi.org/10.4324/9780203019672
Marfuah, M., Suryadi, D., Turmudi, T., & Isnawan, M. G. (2022). Providing online learning situations for in-service mathematics teachers' external transposition knowledge during COVID-19 pandemic: Case of Indonesia. *Electronic Journal of E-Learning, 20*(1), 69–84. https://doi.org/10.34190/ejel.20.1.2388
Matei, S. A., & Britt, B. C. (2017). Organizational configurations and configurational change. In *Structural differentiation in social media* (pp. 97–112). Springer.
McDermott, S. M. (2022). Using technology to teach sustainability with applications to conservation biology and ecosystem service management. In *Teaching environmental and natural resource economics*. Edward Elgar Publishing. https://doi.org/10.4337/9781788114288.00021.
Munroe, A., & Pearson, C. (2006). The munroe multicultural attitude scale questionnaire: A new instrument for multicultural studies. *Educational and Psychological Measurement, 66*(5), 819–834.
Nguyen, T. (2015). The effectiveness of online learning: Beyond no significant difference and future horizons. *MERLOT Journal of Online Learning and Teaching, 11*(2), 309–319.
Oakes, J. (1986). Tracking, inequality, and the rhetoric of reform: Why schools don't change. *Journal of Education, 168*(1), 60–80. https://doi.org/10.1177/002205748616800104

Peng, K. L., Shen, H., & Lin, P. M. C. (2020). Online education philosophy and evolution between regions of Greater China: Impacts of COVID-19. *Tourism Tribune, 35*(5), 6–9.
Poudel, S., Ishak, A., Perez-Fernandez, J., Garcia, E., León-Figueroa, D. A., Romaní, L., Bonilla-Aldana, D. K., & Rodriguez-Morales, A. J. (2022). Highly mutated SARS-CoV-2 Omicron variant sparks significant concern among global experts–What is known so far? *Travel Medicine and Infectious Disease, 45*, Article 102234. https://doi.org/10.1016/j.tmaid.2021.102234
Qu, X., Xie, Y., & Lin, J. (2015). *Quantitative analysis and evaluation of the teaching process based on information entropy. 2015 international conference on social science*. Education Management and Sports Education.
Rasheed, R. A., Kamsin, A., & Abdullah, N. A. (2020). Challenges in the online component of blended learning: A systematic review. *Computers & Education, 144*, Article 103701.
Reich, J. (2020). *Failure to disrupt: Why technology alone can't transform education*. Harvard University Press.
Reimers, F. M. (2022). Learning from a pandemic. The impact of COVID-19 on education around the world. In *Primary and secondary education during covid-19* (pp. 1–37). Springer. https://doi.org/10.1007/978-3-030-81500-4_1.
Rusmono, R., Winarsih, M., & Hardiansyah, H. (2019). Effect of teaching material based on mobile learning to learning outcomes of natural environment. *Journal of Physics: Conference Series, 1402*(7), 077076.
Samson, S. (2010). Information literacy learning outcomes and student success. *The Journal of Academic Librarianship, 36*(3), 202–210.
Sandeen, C. (2013). Integrating MOOCs into traditional higher education: The emerging "MOOC 3.0" era. *Change: The Magazine of Higher Learning, 45*(6), 34–39.
Scherer, R., Siddiq, F., & Tondeur, J. (2019). The technology acceptance model (TAM): A meta-analytic structural equation modeling approach to explaining teachers' adoption of digital technology in education. *Computers & Education, 128*, 13–35.
Serenko, A., Detlor, B., Julien, H., & Booker, L. D. (2012). A model of student learning outcomes of information literacy instruction in a business school. *Journal of the American Society for Information Science and Technology, 63*(4), 671–686.
Shannon, C. E. (1948). A mathematical theory of communication. *The Bell system technical journal, 27*(3), 379–423.
Sheng, W. X., Fu, Q. Y., & Dong, X. R. (2017). Research and application of Information entropy in teaching level questionnaire. *Journal of Nanchang University, 41*(6), 596–599.
Shulla, K., Filho, W. L., Lardjane, S., Sommer, J. H., & Borgemeister, C. (2020). Sustainable development education in the context of the 2030 Agenda for sustainable development. *The International Journal of Sustainable Development and World Ecology, 27*(5), 458–468. https://doi.org/10.1080/13504509.2020.1721378
Sinclair, J., & Kalvala, S. (2016). Student engagement in massive open online courses. *International Journal of Learning Technology, 11*(3), 218–237. https://doi.org/10.1504/IJLT.2016.079035
Sugandi, L., & Kurniawan, Y. (2018). The influence of information technology on the information and service quality for the teaching and learning. *International Journal of Emerging Technologies in Learning (iJET), 13*(12), 230–237.
Taglietti, D., Landri, P., & Grimaldi, E. (2021). The big acceleration in digital education in Italy: The COVID-19 pandemic and the blended-school form. *European Educational Research Journal, 20*(4), 423–441. https://doi.org/10.1177/14749041211021246
Tamrat, S. (2020). *Re-thinking humanism as a guiding philosophy for education: A critical reflection on Ethiopian higher educat0ion institutions*.
Tandon, U., Kiran, R., & Sah, A. N. (2016). Customer satisfaction using website functionality, perceived usability and perceived usefulness towards online shopping in India. *Information Development, 32*(5), 1657–1673.
Tandon, U., Kiran, R., & Sah, A. N. (2018). The influence of website functionality, drivers and perceived risk on customer satisfaction in online shopping: An emerging economy case. *Information Systems and E-Business Management, 16*(1), 57–91.
Tsai, D.-Y., Lee, Y., & Matsuyama, E. (2008). Information entropy measure for evaluation of image quality. *Journal of Digital Imaging, 21*(3), 338–347.
Wang, L. (2007). Sociocultural learning theories and information literacy teaching activities in higher education. *Reference and User Services Quarterly*, 149–158.
Wang, Z.-g. (2007). Impact of information entropy on teaching effectiveness. *Online Submission, 4*(3), 77–80. https://eric.ed.gov/?id=ED497392.
Wang, Z. (2007). Impact of information entropy on teaching effectiveness. *Online Submission, 4*(3), 77–80.
Wang, X., & Li, X. (2003). On transferring effective lnformation in modern classroom teaching. *Journal of Shaanxi Institute of Education, 19*(2).
Xing, J. (2013). *General education and the development of global citizenship in Hong Kong, Taiwan and Mainland China: Not merely icing on the cake*. Routledge.
Yin, H., Wang, W., & Han, J. (2016). Chinese undergraduates' perceptions of teaching quality and the effects on approaches to studying and course satisfaction. *Higher Education, 71*(1), 39–57.

Dr. Kang-Lin Peng is an Associate Professor in the Faculty of International Tourism and Management at the City University of Macau, Macau SAR, China. His research interest includes hospitality financial management and hospitality education (e-mail: klpeng@cityu.mo).

Dr. Pearl M.C. Lin is an Associate Professor in the School of Hotel and Tourism Management at The Hong Kong Polytechnic University, Hong Kong SAR, China. Her research interest includes sharing economy, culinary education and food tourism (e-mail: pearl.lin@polyu.edu.hk).

Dr. Jusi Xu is a Lecturer in the School of Hospitality and Tourism Management at Shunde Polytechnic, Guangdong, China. Her research interest includes hospitality and tourism research (e-mail: j_xu23@outlook.com).

Dr. Xin Wang is an Assistant Professor in the Faculty of International Tourism and Management at the City University of Macau, Macau SAR, China. Her research interest includes Tourism consumer behavior (e-mail: xwang@cityu.mo).
